# Esophago-tracheobronchial fistula induced by anlotinib: A case report

**DOI:** 10.1097/MD.0000000000046215

**Published:** 2025-11-21

**Authors:** Yiheng Qian, Jiabin Qian, Xin Lv

**Affiliations:** a Department of Pulmonary Medicine, The First Affiliated Hospital of Zhejiang Chinese Medical University (Zhejiang Provincial Hospital of Chinese Medicine), Hangzhou, China.

**Keywords:** anlotinib, esophago-tracheobronchial fistula, squamous cell lung carcinoma

## Abstract

**Rationale:**

Anlotinib is a novel multi-target tyrosine kinase inhibitor (TKI) that targets tumor angiogenesis and proliferation signals, demonstrating significant antiangiogenic effects. It is used as a third-line treatment for refractory advanced non-small cell lung cancer.

**Patient concerns:**

An elderly male patient with lung squamous cell carcinoma, who was intolerant to chemotherapy, received anlotinib for antitumor treatment and experienced choking when eating and drinking.

**Diagnoses:**

Pulmonary computed tomography revealed an irregular cavity adjacent to the mediastinum in the right lung, measuring ~75 mm × 49 mm. Endoscopic examination combined with gastroscopy and tracheoscopy under general anesthesia showed erosion of the carina, absence of the right side, and an ~3-cm-long, oval-shaped esophagotracheal fistula located 26 to 29 cm from the incisors.

**Interventions:**

A partially covered esophageal stent was placed endoscopically.

**Outcomes:**

During the 6-month follow-up period, the patient’s esophagotracheal fistula narrowed, and he no longer experienced choking while eating or drinking.

**Lessons:**

The incidence of adverse reactions to anlotinib is relatively low, and esophago-tracheobronchial fistula caused by it is even rarer. When using anlotinib to treat non-small cell lung cancer, clinicians need to monitor changes in patients’ symptoms closely. If symptoms of choking when eating and drinking occur, pulmonary computed tomography should be promptly repeated to detect signs of esophago-tracheobronchial fistula early and intervene promptly.

## 1. Introduction

**Figure 1.**
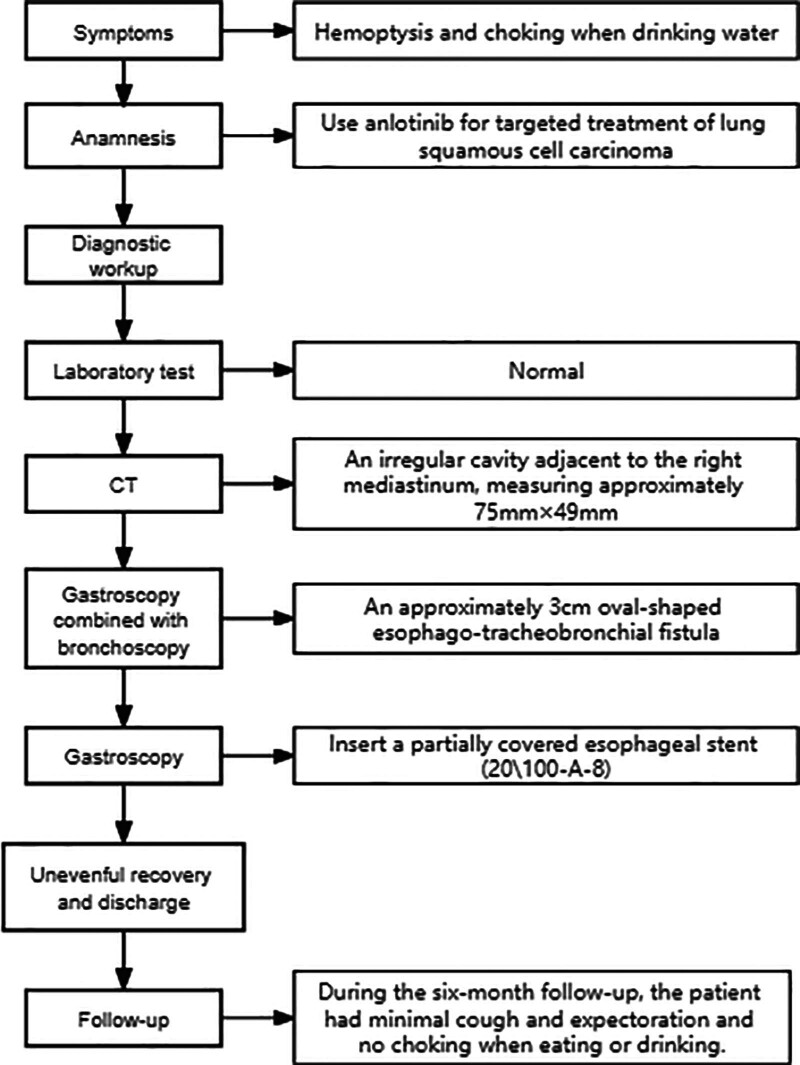
Timeline illustrates the diagnosis and treatment process of the patient.

Anlotinib has a significant antiangiogenic effect. Esophago-tracheobronchial fistula is a rare complication during the treatment with anlotinib, mainly manifested as coughing while drinking, difficulty in swallowing, recurrent aspiration pneumonia, and malnutrition. The prognosis is poor. Even with active treatments such as discontinuation of medication and placement of stents, there may still be a fatal outcome. We report the case of a 79-year-old patient with advanced squamous cell lung cancer who developed esophago-tracheobronchial fistula after using anlotinib due to intolerance to chemotherapy. The patient experienced coughing when drinking water 1 month after starting anlotinib and was diagnosed with esophago-tracheobronchial fistula. After the placement of a partially covered esophageal stent under endoscopy, the symptoms improved (Fig. 1).

## 2. Case presentation

**Figure 2.**
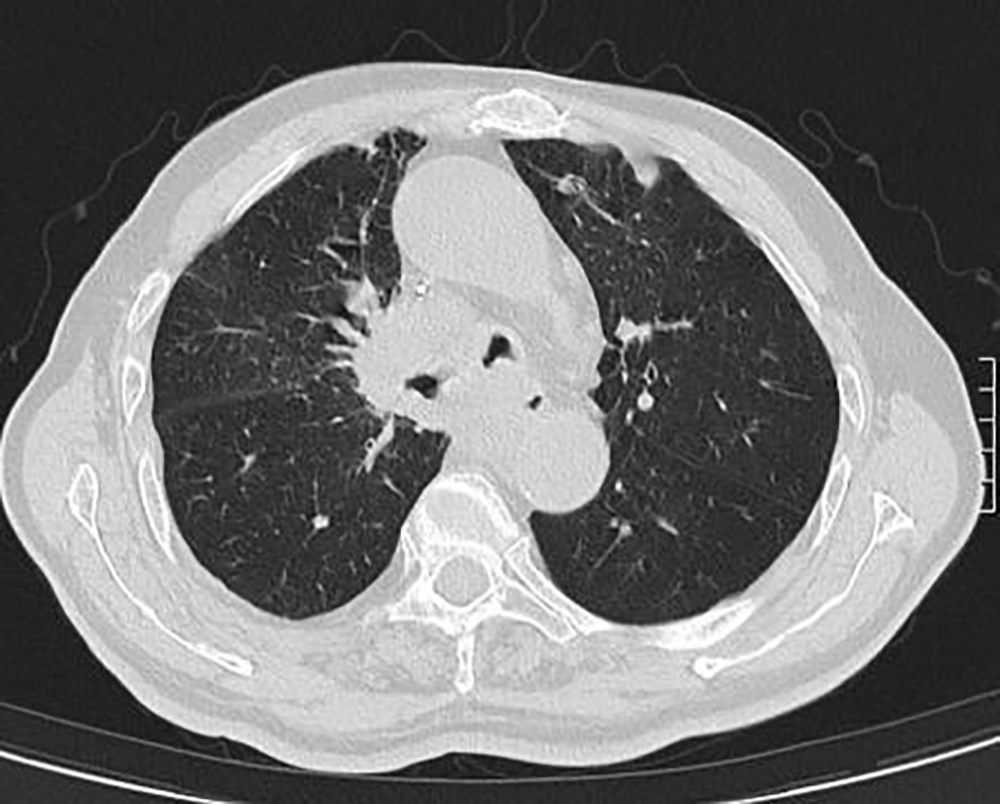
A soft tissue mass is observed in the right upper pulmonary hilum, the lower segment of the trachea, and the area surrounding the carina. Local stenosis is noted in the left and right main bronchi and the esophagus.

**Figure 3.**
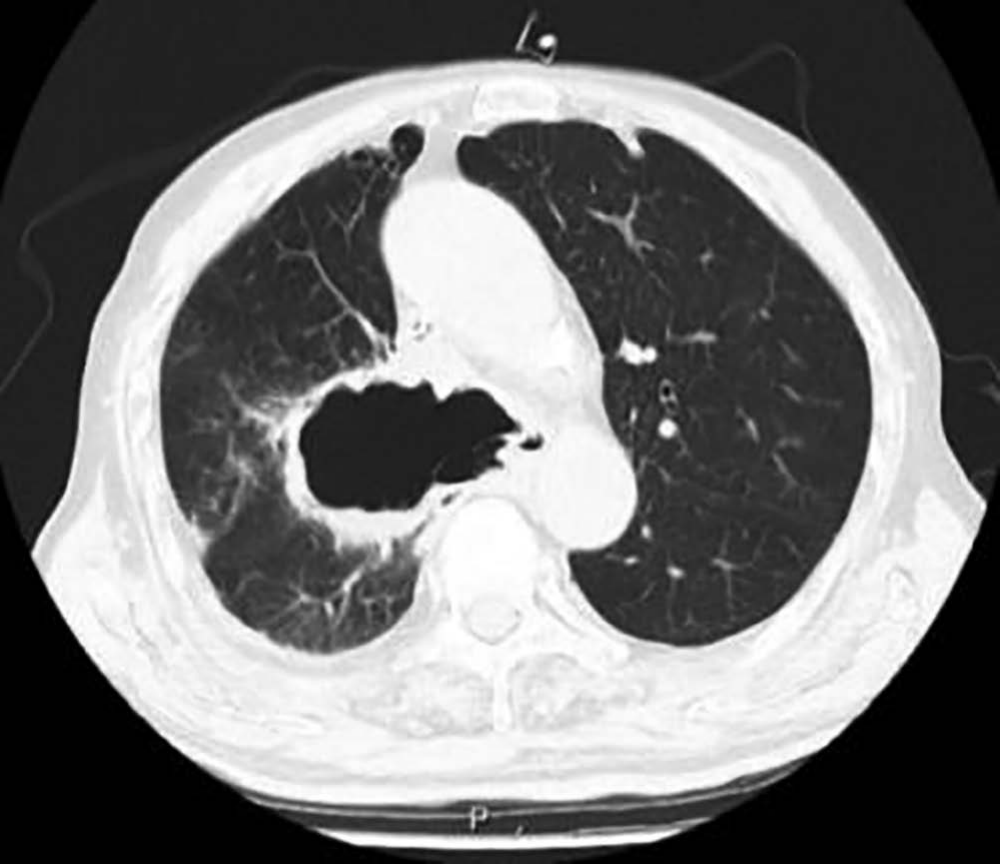
An irregular cavity shadow is observed in the mediastinum of the right lung, with a size of ~75 mm × 49 mm in the horizontal section.

**Figure 4.**
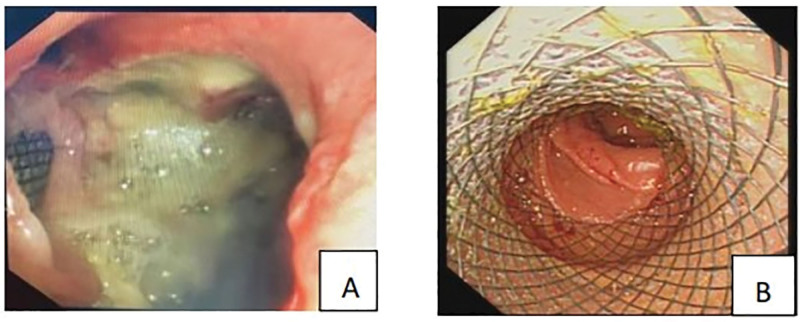
(A) It shows tracheal carina erosion, missing on the right side, and an oval-like esophagotracheal fistula with a length of about 3 cm in diameter. (B) It shows the fistula after insertion of an esophageal semi-grafted stent.

**Figure 5.**
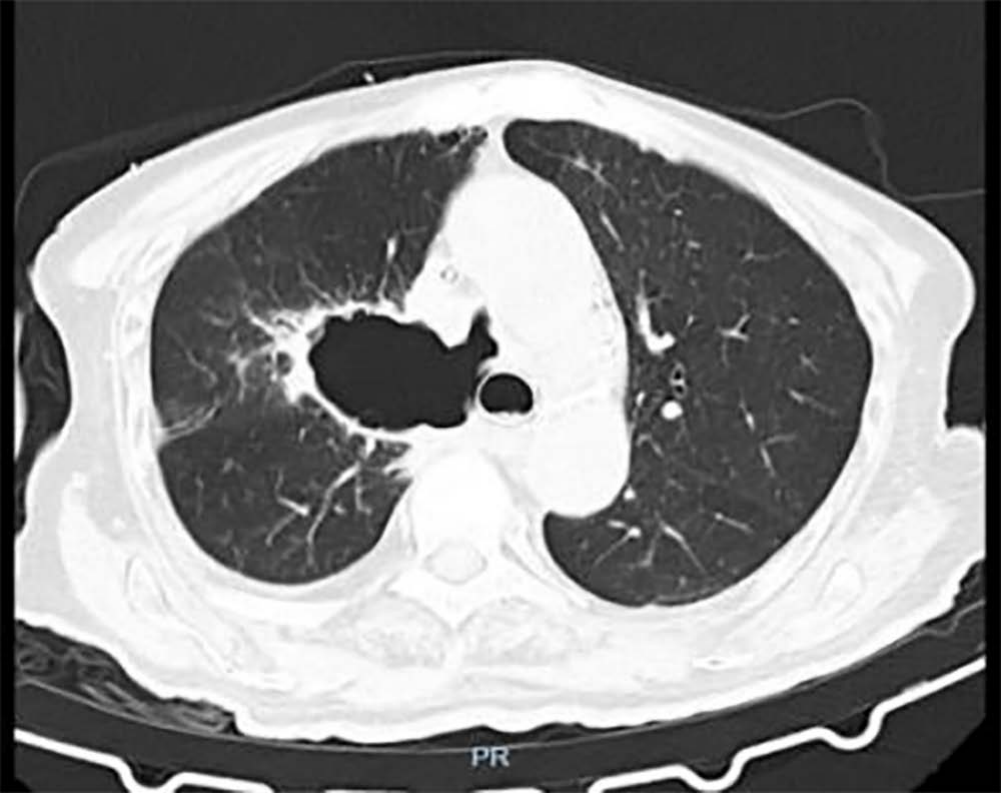
The irregular cavity shadow has shrunk to 72 × 45 mm.

A 79-year-old male patient was admitted to the First Clinical Hospital Affiliated to Zhejiang Chinese Medical University on September 5, 2024, due to “confirmed lung squamous cell carcinoma for 3 years, blood in sputum for 3 months, and choking cough for 1 month.” The patient had a 40-year smoking history. Three years ago, he was diagnosed with stage cT4N1M1a IVA lung squamous cell carcinoma at another hospital. After undergoing 5 cycles of paclitaxel and nedaplatin chemotherapy, he experienced severe vomiting and loss of appetite, leading to the cessation of systemic chemotherapy and the initiation of Chinese herbal medicine treatment. Three months prior, he developed a cough with expectoration of bloody sputum. A pulmonary computed tomography (CT) scan (Fig. 2) showed lung occupation with multiple enlarged mediastinal lymph nodes and local narrowing of the left and right main bronchi and esophagus. A bronchoscopy performed at another hospital revealed new growths in the lower trachea, the openings of the left and right main bronchi, and the carina, with mucosal infiltration and luminal narrowing. The patient refused further treatments such as chemotherapy and was prescribed anlotinib 12-mg QD orally for 14 days. One month ago, the patient experienced increased hemoptysis and a choking cough when drinking water. A pulmonary CT scan (Fig. 3) revealed an irregular cavity adjacent to the right mediastinum, measuring ~75 mm × 49 mm, with partially irregular thickening of the walls, fluid levels inside, partial communication with the bronchi, unclear structure of the right main bronchus, and mild-to-moderate enhancement of the cavity walls after contrast administration. The area adjacent to the esophagus showed unclear local structures. On September 11, 2024, a combined gastroscopy and bronchoscopy under general anesthesia (Fig. 4) revealed erosion of the carina, with the right side missing. An ~3-cm oval-shaped esophago-tracheobronchial fistula was observed between 26 and 29 cm from the incisors, with a large amount of viscous food residue and mucus retained in the fistula sac. After removing necrotic tissue from the fistula, a partially covered esophageal stent (20\100-A-8) was placed. The day after the surgery, the patient’s symptoms of cough, expectoration, and hemoptysis improved, and he could eat without choking. One week post-surgery, a follow-up pulmonary CT scan (Fig. 5) showed the irregular cavity adjacent to the right mediastinum with no significant air-fluid level and a slightly thinner local cyst wall. Four months later, a follow-up pulmonary CT scan showed a reduction in the size of the fistula, but the patient experienced massive hemoptysis, which improved after bronchial artery embolization. During the 6-month follow-up, the patient had minimal cough and expectoration and no choking when eating or drinking.

## 3. Discussion

Esophago-tracheobronchial fistula refers to an abnormal communication between the esophagus and trachea, allowing the exchange of gas and fluid between the digestive tract and the airway.^[1,2]^ Colonized bacteria in the digestive tract can migrate to the respiratory tract through the fistula, causing bacterial inflammation. The occurrence of esophago-tracheobronchial fistula is often related to various factors such as malignant tumors, infections, and trauma, and is one of the common complications of esophageal cancer. Esophago-tracheobronchial fistula makes oral intake difficult, leading to poor nutritional status and persistent respiratory infections.^[3]^ Placement of metal-covered or silicone stents under bronchoscopic guidance is an effective method for treating esophago-tracheobronchial fistula,^[2]^ which can effectively seal the fistula but cannot prevent stent migration.^[4]^ In this case, the patient’s lung condition progressed significantly in June 2024, with narrowing of the trachea and bilateral bronchi, and infiltration changes in the esophagus. Anlotinib was administered for 1 course starting in July. In August, the patient experienced repeated choking cough while eating and drinking, and a pulmonary CT scan showed a large and irregular cavity adjacent to the right mediastinum. To further confirm the diagnosis, we performed a combined gastroscopy and bronchoscopy, which revealed an ~3-cm oval-shaped fistula located ~26 to 29 cm from the incisors. A partially covered esophageal stent was then placed. Using the Naranjo scale, we assessed the score as 4. After discharge, anlotinib was not continued, and the patient did not experience a choking cough while eating or drinking during the 6-month follow-up.

Anlotinib is a novel multi-target tyrosine kinase inhibitor that targets tumor angiogenesis and proliferation signaling pathways, achieving tumor suppression through multiple mechanisms: it inhibits the vascular endothelial growth factor receptor 2/3-mediated signaling pathway by blocking the phosphorylation of vascular endothelial growth factor receptor 2/3 and it exhibits strong inhibitory effects on platelet-derived growth factor receptor α/β and fibroblast growth factor receptor 1 to 4 pathways.^[5]^ The vascular endothelial growth factor family is the most important and typical molecular factor promoting malignant tumor angiogenesis,^[6,7]^ playing a crucial role in tumor growth and metastasis through angiogenesis.^[8]^ In Phase III clinical trial, the median overall survival (OS) and progression-free survival were significantly increased in the anlotinib group, with significant improvements in both the objective response rate and disease control rate (DCR). Binyan Lin^[9]^ in both rat aortic ring assay and chicken chorioallantoic membrane assay could simulate the process of angiogenesis in vivo, further confirming that anlotinib inhibits vascular bundle germination and reduces microvascular density and in wound healing assay, chamber directional migration assay, and tube formation assay; anlotinib significantly inhibits cell migration and tube formation in endothelial cells, which are the crucial steps for neovascularization. In this case, the patient with left lung squamous cell carcinoma was intolerant to chemotherapy. Bronchoscopy revealed new growths and mucosal infiltration in the lower trachea, the openings of the left and right main bronchi, and the carina, providing an anatomical and pathological basis for the formation of an esophago-tracheobronchial fistula. Due to the antiangiogenic effects of anlotinib, there is also a risk of bleeding, and it is contraindicated in principle for patients with central lung squamous cell carcinoma or those at high risk of massive hemoptysis. Therefore, the use of anlotinib for antitumor treatment may reduce the angiogenic and reparative capacity of the esophageal and tracheal mucosa, leading to tissue ischemia, necrosis, and subsequently promoting the formation of an esophago-tracheobronchial fistula. After the patient used anlotinib, the tumor lesion significantly shrank, but a pulmonary cavity combined with a large esophago-tracheobronchial fistula occurred. Other studies have shown that the formation of pulmonary cavities is related to the efficacy of anlotinib: among 96 patients, 12.5% developed pulmonary cavities, with a DCR of 100%, suggesting that patients in the lesion cavitation group had better short-term efficacy compared with the overall population (DCR 85%). However, short-term efficacy does not represent overall OS, and the median OS of patients with pulmonary cavities was significantly shorter than that of patients without pulmonary cavities, indicating that lesion cavitation may be associated with poorer OS.

Endoscopic placement of covered tracheal and/or esophageal stents is an important treatment method for esophago-tracheobronchial fistulas, which can promote fistula healing, control pulmonary infections,^[10]^ improve quality of life, extend survival time, and demonstrate good safety. Uncovered metal bare stents allow rapid growth of tumor tissue to block the mesh, leading to issues such as stent retrieval difficulty, bleeding, and perforation. Wang^[3]^ achieved good results using airway metal-covered stents to seal tracheoesophageal fistulas of different locations and sizes caused by malignant tumors. Kim^[10]^ demonstrated that a single esophageal stent is suitable for malignant esophago-tracheobronchial fistulas without airway stenosis or airway compression. Yuya Nishio^[11]^ also proposed the use of a “double-stent placement” technique using 2 metal stents of the same size (1 uncovered and 1 fully covered) to treat esophago-tracheobronchial fistulas and correct initial stent migration. The uncovered stent and the fully covered stent are fixed together by horizontal frictional force. The fully covered stent does not cause stenosis due to tumor growth within the stent lumen, but it has a lower coefficient of friction compared with the uncovered stent and is prone to migration from the original implantation site.

To gain a comprehensive understanding of esophago-tracheobronchial fistulas caused by anlotinib, as previously reported, a literature search was conducted using the keywords “anlotinib” and “esophago-tracheobronchial fistula” in the Wanfang Database, VIP Database, and CNKI, but no relevant articles were found. A search was then performed in the PubMed database using “esophago-tracheobronchial fistula” and “anlotinib” as All Fields, which yielded 2 articles. One of these articles did not contain relevant cases, so only 1 article was included, reporting 2 cases of esophago-tracheobronchial fistulas induced by anlotinib. One case involved a 55-year-old nonsmoking female patient with squamous cell carcinoma, and the other case involved a 53-year-old nonsmoking male patient with squamous cell carcinoma. After receiving 6 cycles of chemoradiotherapy, both patients were treated with oral anlotinib (12-mg QD) on days 1 to 14 of a 21-day cycle due to lung progression. The cough developed 1 month after starting the medication, which worsened after swallowing. Esophagoscopy confirmed the diagnosis of esophago-tracheobronchial fistula, and a fully covered self-expanding metal stent was placed, resulting in relief of cough without stent migration, bleeding, or secondary fistulas. Both patients subsequently died of lung cancer metastasis.

In this case, bronchoscopy revealed erosion of the carina, absence on the right side, significant mucosal congestion at the opening of the left main bronchus, local granular mucosal changes, enlargement of the right main bronchial lumen with unclear structure, and a large esophago-tracheobronchial fistula with a large amount of yellow-green food residue covered with white moss. Therefore, it was not possible to place a tracheal stent, and a semi-covered esophageal stent was implanted instead. Semi-covered stent is superior to full-covered stent in lumen compliance and conformability. Sufficient tension generated by the stent enables esophageal mucosal tissue to protrude into the stent through these small holes, which plays a role in fixing the stent, thus improving the disadvantage that the covered stent is easy to slip off. On the day after the surgery, the patient did not experience coughing or choking when eating or drinking. A follow-up pulmonary CT 4 months later showed a reduction in the fistula size. During the 6-month follow-up period, there was no recurrence of coughing or choking.

For non-small cell lung cancer patients treated with anlotinib, the most common adverse reactions are hypertension, hyponatremia, and elevated gamma-glutamyl transferase.^[12]^ For patients with advanced non-small cell lung cancer receiving anlotinib therapy, regular follow-up pulmonary CT scans are necessary. If symptoms such as coughing while eating or drinking occur, the possibility of esophago-tracheobronchial fistula should be promptly considered and addressed as early as possible.

## 4. Conclusion

Esophago-tracheobronchial fistula caused by anlotinib is rare, and its pathogenesis remains unclear, necessitating further in-depth exploration by scholars. Coughing while drinking or swallowing is one of the characteristic clinical manifestations of esophago-tracheobronchial fistula. When these symptoms occur, it is necessary to screen for esophago-tracheobronchial fistula. The diagnosis mainly relies on pulmonary CT, bronchoscopy, etc. Endoscopic placement of tracheal and/or esophageal covered stents is currently the preferred method for treating esophago-tracheobronchial fistula.

## Acknowledgments

We are grateful to the patient for agreeing to post his information and to all the medical staff.

## Author contributions

**Writing – original draft:** Yiheng Qian.

**Writing – review & editing:** Yiheng Qian, Jiabin Qian, Xin Lv.
